# Improving Immune Checkpoint Therapy in the Tumor Microenvironment by Targeting T Cell Metabolism

**DOI:** 10.2174/0118715303400871250821103310

**Published:** 2026-09-02

**Authors:** Jun Wei, Baozhen An, Renchao Dong, Xinyi Tu, Yifei Dong, Yuang Liu, Yanqiu Liu

**Affiliations:** 1 *Shandong University of Traditional Chinese Medicine, Jinan, China; *; 2 *The First Affiliated Hospital of Shandong University of Traditional Chinese Medicine, Jinan, China*

**Keywords:** Tumor microenvironment, nutrient competition, T cell metabolism, immune checkpoint, tumor immunotherapy, combination therapy

## Abstract

To meet the increased nutrient requirements associated with rapid cellular proliferation, tumor cells undergo metabolic reprogramming, characterized by a substantial increase in the production of energy and precursor molecules necessary for biosynthetic processes. Similarly, T cells experience metabolic reprogramming to support their proliferation and immunological functions, leading to metabolic competition with tumor cells within the tumor microenvironment (TME). This metabolic competition adversely affects T cell activation, proliferation, and immune function, primarily due to the limited availability of glucose, lipids, and amino acids. Furthermore, cytokines and immune checkpoints significantly impact T cell-mediated immunoreactivity. Modulating the metabolism of tumor cells and T cell-mediated immune evasion within the TME is of paramount importance. Notably, the metabolism of small-molecule target agents has garnered considerable attention in the context of the TME. This study aimed to examine the influence of various microenvironmental factors on T cell metabolism and explore corresponding innovative therapeutic approaches, thereby offering a comprehensive array of potential clinical strategies for cancer prevention and treatment.

## 1 INTRODUCTION

The incidence and mortality of tumors are increasing yearly, making it one of the most prominent public health problems in recent years [1, 2]. Tumor tissues are more than just a collection of cancer cells; they also contain a complex tumor microenvironment (TME) that influences tumor development and progression [3, 4]. The progression of tumorigenesis is influenced by genetic or epigenetic alterations as well as rearrangements within the TME [5]. Composed of extracellular matrix, stromal cells, immune cells, and other components, the TME plays a critical role in regulating tumor cells proliferation and metastasis [6, 7]. Recent studies have highlighted the pivotal role of the microbiome in the TME, including the micro- and mycobiome. Accumulating evidence suggests that local microbiota is integral to the TME across various cancer types, particularly those originating from mucosal sites, such as the lung, skin, and gastrointestinal tract [8, 9].

Tumor cells modulate the functions of both cellular and non-cellular components within the TME* vi*a an intricate signaling network. This network fosters tumor cells heterogeneity and enhances drug resistance [10, 11]. The substantial nutrient and precursor molecule requirements of tumor cells underpin their rapid proliferation and metastatic potential [12]. In response, tumor cells reprogram their metabolism, primarily altering glucose, amino acid, and lipid metabolic pathways [13-15]. Tumor cells exhibit a high rate of glycolysis, producing copious amounts of lactic acid even under aerobic conditions, which is referred to as the Warburg effect [16]. Elevated glutamine metabolism serves as a primary energy source, surpassing normal biosynthetic and metabolic demands. The unique metabolic pattern creates an acidic, hypoxic, and nutrient-deprived tumor microenvironment, which shapes the immunosuppressive efficacy [17-19].

T cells represent the most crucial immune component within the TME, mediating anti-tumor immunity primarily through interactions between CD4^+^ and CD8^+^ T cells and tumor-associated antigens [20]. Both tumor cells and T cells undergo significant metabolic reprogramming within the TME, which can suppress T cell function and promote tumor progression [21, 22]. Inhibiting T cell metabolism may impair their ability to produce interferon-gamma (IFN-γ), thereby diminishing anti-tumor immunity. Consequently, modulating metabolic pathways to restore T cell function may enhance the efficacy of anti-tumor immunotherapy.

This study focused on the metabolic characteristics of T cells in the TME. Changes in nutrient availability affect T cell proliferation and activation, as well as their immune function in the tumor microenvironment. Furthermore, the immunomodulatory effects and mechanisms of tumor metabolites, various cytokines, and immune checkpoints on T cells in the TME were discussed. We also summarized the metabolism and mechanisms of various microenvironmental factors on T cells, as well as the corresponding metabolic immunomodulatory agents. The findings provide potential molecular targets and novel clinical therapeutic strategies for the prevention and treatment of tumors.

In this review, we searched related articles from the PubMed database. We used combinations of keywords related to T cell metabolism, immune checkpoint, tumor microenvironment, nutrient competition, tumor immunotherapy, combination therapy, and CAR T cells. The search was restricted to terms appearing in article titles or abstracts to ensure relevance. After content screening and investigation of the research background, a total of 141 articles were finally included in this review.

## 2 THE METABOLIC CHARACTERISTICS OF T CELLS

The metabolic patterns of T cells are intricately linked to their functional states, particularly within the TME [23, 24]. Recent research has highlighted that T cells exhibit distinct metabolic profiles depending on their activation and differentiation states, which can significantly influence their ability to respond to tumors. For instance, activated T cells predominantly rely on glycolytic metabolism to support proliferation and effector functions, whereas exhausted or dysfunctional T cells often exhibit limited metabolic capacity, which hinders their effectiveness in combating tumors [25]. The emergence of an adaptive immune response depends on the proliferation and differentiation of early T cells that detect specific antigens in large numbers over a short period. Subsequently, most antigen-specific T cells are cleared *via* apoptosis, while a few develop into memory T cells [26]. Different T cell subsets utilize metabolic pathways, such as glycolysis, oxidative phosphorylation (OXPHOS), and fatty acid oxidation (FAO), to adapt to the nutritional levels and energy demands of the TME (Fig. **1**) [27-29] .

Naive T cells exhibit low metabolic activity, relying primarily on fatty acid oxidation and oxidative phosphorylation to maintain normal physiological functions [30]. When T cell receptors (TCRs) recognize antigens and receive co-stimulatory signals from antigen-presenting cells (APCs), T cells expand and function as effector cells, causing changes in metabolic pathways [31]. To support the higher metabolic demands, activated T cells upregulate nutrient transport proteins, increasing the uptake of glucose and amino acids. The main pathway of energy generation shifts from low levels of oxidative phosphorylation to high levels of glycolysis and glutamine metabolism [32]. Notably, glycolysis exerts a vital role in maintaining the functional activity of T cells, which is required for T cell production of IFN-γ. In addition, aerobic glycolysis is closely associated with the expression degree of programmed cell death protein-1 (PD-1), which may alter the efficacy of tumor immunotherapy [33].

## 3 IMMUNE DISORDERS STEMMING FROM METABOLIC DEFICIENCIES IN T CELLS

The metabolic characteristics of T cells are crucial for their function and can vary significantly across different T cell types, influencing their role in health and disease. Regulatory T cells (Treg cells) are essential for maintaining immune tolerance and preventing autoimmune diseases. The metabolic fitness of Tregs is orchestrated by signaling pathways, such as LKB1, which is crucial for their survival and function, independent of conventional AMPK signaling. This highlights the unique metabolic adaptations that Tregs undergo to maintain immune homeostasis and prevent inflammatory diseases [34]. In contrast, effector T cells (Teffs), such as CD8^+^ cytotoxic T lymphocytes, undergo significant metabolic reprogramming upon activation. However, in the tumor microenvironment, these cells often face metabolic challenges due to nutrient depletion and hypoxia, which can impair their function. Strategies to restore their metabolic capacity, such as targeting bioenergetic pathways, are being explored to enhance their anticancer activity [35]. Understanding these metabolic characteristics and disorders across different T cell types can provide insights into therapeutic strategies for various diseases, including cancer and autoimmune disorders.

Currently, single-cell RNA sequencing (scRNA-seq) has emerged as a powerful tool for unraveling the complexity of the TME, particularly in identifying distinct subsets of T cells with unique metabolic characteristics. Recent studies have utilized scRNA-seq to uncover the metabolic heterogeneity among T cell subsets in various cancers. For instance, in melanoma, scRNA-seq analysis has identified several CD8^+^ T cell subpopulations, each exhibiting distinct metabolic profiles. Notably, exhausted CD8^+^ T cells were found to exhibit abnormal activity in metabolic pathways, which may contribute to their impaired function in the TME [36]. Similarly, in clear cell renal cell carcinoma, scRNA-seq revealed that the energy metabolism of CD8^+^ T cells is crucial for their cytotoxic effector functions and serves as a marker of impending cellular exhaustion [37]. Regulating autophagy in CD8^+^ T cells is crucial for their function and survival. ATG5 deficiency hinders T cell growth and increases cell death, indicating its importance in immune responses [38]. FIP200 has dual roles: its autophagy function facilitates tumor growth, while its non-canonical role enhances T-cell infiltration and improves immune checkpoint therapy in breast cancer [39]. Targeting autophagy regulators, such as ATG5 and FIP200, could enhance cancer immunotherapy by improving CD8^+^ T cell survival and effectiveness, potentially countering immunosuppressive tumor environments [40].

## 4 T CELL METABOLISM IN TUMOR PROGRESSION

Tumor cells and T cells undergo modifications in extensive metabolic pathways in response to stimuli from the tumor microenvironment, resulting in the suppression of T cells and the promotion of tumor progression [41-43]. The effector functions of T cells are activated by specialized metabolic pathways, which also regulate the trajectory of T cell differentiation. Specifically, naive T cells predominantly utilize oxidative phosphorylation, Teffs rely on glycolysis, and regulatory and memory T cells primarily engage in lipid oxidation metabolic pathways (Fig. **2**) [41].

The effects of TME on T cell metabolism are multifaceted, involving reprogramming of glucose, amino acid, and lipid metabolism. This metabolic reprogramming not only supports the rapid proliferation of tumor cells but also affects the function of T cells by competing for energy resources and changing the microenvironment [44]. For example, aerobic glycolysis in tumor cells consumes glucose, leading to lactic acid accumulation, which in turn inhibits T cell activity [45]. Additionally, changes in lipid metabolism can alter the composition of cell membranes and disrupt signal transduction, thereby weakening T cell function [46]. Reprogramming of amino acid metabolism, such as increased consumption of glutamine and arginine by tumor cells, further impedes T cell activity and proliferation [47].

### 4.1 Glucose Metabolism

As an essential energy source for tumors and T cells, glucose rapidly provides ATP and metabolic intermediates *via* the glycolytic route [48]. Glycolysis is a critical pathway for energy production in cancer cells, and the enzyme hexokinase 2 (HK2) plays a pivotal role in this process. HK2 is often overexpressed in cancer cells, facilitating the conversion of glucose to glucose-6-phosphate, which is the first step in glycolysis. This enzyme's activity is linked to the transformation of glycolysis from oxidative OXPHOS to aerobic glycolysis [49]. Tumors and T cells exhibit a similar relationship to aerobic glycolysis, which may compete for nutrients in the TME [50, 51]. The enhanced aerobic glycolysis of tumor cells reduces the glucose content of the tumor microenvironment. Changes in tumor cell glucose metabolism impair the capacity of T cells to eliminate tumors, thereby reducing anti-tumor immunity [52].

Tumor cells utilize more glucose than immune cells, which reduces the glucose uptake by T cells and impacts effector T cell activity. As reported in a study [53], the deletion of glucose reduced the glycolytic capacity of T cells. Furthermore, glycolytic inhibition reshaped the balance between Teffs and Tregs through modulation of the AMPK/mTOR pathway. In addition, GLUT1 was highly expressed on the surface of tumor-infiltrating Teffs surface and higher levels of glycolysis-related genes [31, 54]. A low concentration of glucose promoted apoptosis of T cells by inducing and activating the pro-apoptotic Bcl-2 family protein BCL2-associated X protein (Bax) and enhancing the instability of anti-apoptotic Bcl-2 family proteins [55]. The reduction in glucose uptake reduced the activity of the mTOR pathway, and glycolytic capacity promoted tumor progression [56]. Nonetheless, when combined with enough glucose, T cell function was greatly enhanced. In contrast, glucose elimination may stimulate PD-1 expression and binding of T cell PD-1 and tumor cell PD-1 ligand-1 (PD-L1), which directly suppresses T cell-mediated immune activity [57].

### 4.2 Amino Acid Metabolism

The activation, proliferation, differentiation, and immunological surveillance are all significantly reliant on amino acid supply [58]. Stimulated and activated by growth factors, T cells enhance amino acid uptake and accelerate nucleotide and protein synthesis, and ATP production to accommodate their rapid proliferation by regulating the expression of amino acid transporters [59]. Tumor cells inhibit the function of immune cells by competing with them for specific amino acids and also promote immune evasion of tumor cells by expressing specific amino acid metabolites that promote the expression of immune evasion-related proteins [60]. Several immunosuppressive factors, including a deficiency in amino acids, collaborate to reduce T cell immunological activity within the tumor microenvironment.

As a semi-essential amino acid, arginine plays a vital role in the activation and control of T-cell immunity [61]. After activation, T cells need to consume L-arginine for anabolism and rapid proliferation, and the main metabolic mode of T cells changes from glycolysis to OXPHOS to ensure T cell survival. Cytotoxic T cells are sensitive to changes in local amino acid utilization, and their anti-tumor activity is significantly decreased when arginine is locally limited. Arginine is rapidly metabolized by macrophages within the tumor, generating an arginine-deficient state in the TME [62]. Furthermore, myeloid-derived suppressor cells (MDSCs) secrete arginase I, which increases the metabolism of L-arginine and degrades arginine, resulting in a lack of arginine in T cells and thereby inhibiting the antitumor activity of T cells in TME [63]. The addition of arginine to the culture medium promotes T cell oxidative phosphorylation, which regulates T cell glutamine metabolism and the degree of chromatin methylation, potentially inducing the formation of additional memory T cells [64]. The conversion of arginine to spermidine by dendritic cells stimulates the production of indoleamine 2, 3-dioxygenase (IDO) and ultimately improves tumor immune escape by reducing the uptake of arginine by T cells and impelling immune cell function through nitric oxide (NO) [65].

Tumor cells overexpress the tryptophan metabolizing enzymes IDO and tryptophan-2,3-dioxygenase (TDO) to metabolize tryptophan to kynurenine (KYN), resulting in low levels of tryptophan in the tumor microenvironment [66]. IDO accelerates the metabolism of tryptophan in the tumor microenvironment. T cells undergo apoptosis due to tryptophan depletion in the TME. Kynurenine [67], when combined with the aryl hydrocarbon receptor (AHR), may enhance Tregs development, decrease dendritic cell activity, and limit the function of effector T cells. Additionally, activation of AHR upregulates PD-1 expression in CD8^+^ T cells, thereby further promoting the immune evasion of tumor cells [68, 69]. IDO inhibits innate and adaptive immune cell responses by depleting tryptophan in the tumor microenvironment, a process that is essential for T cell proliferation. The accumulation of tryptophan metabolites and their impaired clearance contribute to immune evasion, promoting tumor cell survival and rapid proliferation.

Glutamine provides amino acids through the conversion of other amino acids, promotes the tricarboxylic acid cycle, provides macromolecular intermediates required for the rapid growth and proliferation of tumor cells, and meets the biological needs of tumor cells [70]. Activated T cells up-regulate the expression of amino acid transporters by activating metabolic regulatory factors, such as C-MYC, and convert glutamine to glutamate by glutaminase [71]. Glutamine deficiency inhibits T cell activation and differentiation, which in turn suppresses the immune function of T cells [72]. In a study, it was found that when glutamine levels in TME were reduced, the tumor-killing potential of CD8^+^ T cells was also significantly reduced. PD-1 expression was down-regulated, and Ki-67-positive cells were increased, suggesting that inhibiting glutamine metabolism could prevent CD8^+^ T cell depletion *in vivo* [73]. The differentiation of T helper type 1 cells (Th1) was significantly inhibited by glutamine deprivation, promoting the differentiation of Foxp3^+^ regulatory T cells [74]. In addition, while exogenous glutamine supplementation did not enhance glutamine absorption by T cells, it did increase the fraction of CD8^+^ T cells and improve cytotoxic capability [75].

Many other amino acid metabolisms have an impact on tumors [76]. To satisfy their own metabolic needs, proliferating T cells need intracellular serine absorption *via* the SLC38A1 and SLC1A5 transporters [77]. Additionally, the depletion of alanine reduces protein synthesis within T cells in the tumor microenvironment, which in turn regulates the activation of immature and memory T cells [78, 79]. As previously documented [80], the uptake of methionine enhances T cell activation by increasing S-adenosyl methionine (SAM) content and histone and RNA methylation. Low levels of SLC43A2 expression in tumor-infiltrating effector CD8^+^ T cells result in low levels of methionine and SAM in T cells, weakening T cell survival and function. As a result, additional amino acid metabolism is crucial for influencing T cell antitumor activity.

### 4.3 Lipid Metabolism

Lipids, being one of the primary nutrients, primarily consist of fatty acids, triglycerides, cholesterol, and phospholipids [81]. Fatty acids are the main source of energy for cellular metabolism and the building blocks of phospholipids and glycolipids in cell membranes, which exerts a vital role in influencing the differentiation and function of immune cells [82, 83]. As the precursor of estrogen and testosterone, cholesterol also plays a role in promoting most cancer progression [84]. Furthermore, cholesterol increases CD36 expression in CD8^+^ T cells; however, the accumulation of intracellular lipid peroxides leads to iron-dependent cell death, which limits the activity of CD8^+^ T cell effectors in tumors and reduces their anti-tumor ability within the tumor environment [85].

Dysregulation of fatty acid metabolism is considered a hallmark of malignant transformation in many kinds of cancer, and it also provides energy supplementation for Tregs in TME. Moreover, fatty acid accumulation within tumor-infiltrating Tregs and the promotion of fatty acid synthesis facilitate the spread of Tregs within the tumor, thereby inhibiting T cell anti-tumor activity [86]. In particular, Tregs maintain rapid growth through increased fatty acid uptake mediated by oxidative phosphorylation of fatty acids. Teffs promotes lipid biosynthesis, and Tm reduces lipid synthesis and promotes FAO [87]. Fatty acid oxidation inhibitors slow the growth of breast cancer in obese mice by enhancing CD8^+^ T cell activity. Enhancing the rate of fatty acid and oxidative metabolism in CD8^+^ T cell mitochondria promotes the production and differentiation of memory CD8^+^ T cells, exhibiting anti-tumor effects in animal models [88]. Macrophages exert a significant immunomodulatory influence within the tumor microenvironment, particularly through the process of FAO. They enhance the expression of carnitine palmitoyl transferase 1α (CPT1α) *via* activation of the peroxisome proliferator-activated receptor-γ signaling pathway, facilitating increased fatty acid uptake and metabolism. This metabolic adaptation not only enhances the immunosuppressive capabilities of macrophages but also contributes to the induction of Tregs. Consequently, this process suppresses the activation of effector T cells and diminishes anti-tumor immune responses [89, 90]. The increased expression of CPT1α is closely related to the proliferation and activation of Tregs, which may be achieved by promoting the uptake and utilization of fatty acids [91, 92].

ATP-citrate lyase (ACLY) is a significant enzyme. ACLY links carbohydrate metabolism to lipid synthesis by converting citrate to acetyl-CoA, a precursor for fatty acid and cholesterol biosynthesis. The activity of this enzyme is frequently upregulated in cancer cells, facilitating lipid accumulation and thereby supporting the rapid growth and proliferation of malignant cells [93]. In endometrial cancer, for instance, ACLY is upregulated, promoting cell proliferation and colony formation while attenuating apoptosis. The regulation of ACLY by obesity-related factors *via* Akt-mediated phosphorylation further underscores its role in cancer metabolism [94].

## 5 INTERACTIONS BETWEEN TUMOR CELLS AND T CELLS IN THE TME

The competitive metabolic pattern of tumor cells results in nutrient depletion in the TME, which leads to compromised T cell activation, proliferation, differentiation, and immune function [95, 96]. The particular metabolic pattern of tumor cells, as well as metabolites and immunological checkpoints, can affect T cell-mediated immune activity by regulating cellular energy metabolic pathways (Fig. **3**) [97].

### 5.1 HIF-1α

As a crucial regulatory transcription factor, hypoxia-inducible factor 1α (HIF-1α) plays a significant role in the survival, proliferation, and invasion of tumor cells under hypoxic conditions [98]. HIF-1α, as a critical factor, enhances tumor resistance to numerous anti-tumor immune responses in most solid tumors [99].

Due to the vascular dysfunction in tumors and the high level of depletion caused by tumor cell activity, the oxygen concentration of the microenvironment is decreased [100]. Activated T cells also use HIF-1α signaling to switch to the glycolytic pathway via* mT*ORC1 and participate in glycolytic reactions downstream of mTOR expression [101]. In addition, HIF-1α inhibits the expression of Glut1, hexokinase, and lactate dehydrogenase, thereby decreasing the level of glycolysis and regulating the proliferation and differentiation of Th17 cells. HIF-1α-mediated T-cell responses to oxygen content also promote glucose uptake and catabolism, synthesize Teffs-secreted cytokines, and facilitate effective activation and clonal proliferation of Teffs [102]. Importantly, HIF1α increases the expression of forkhead box P3 (FoxP3) and PD-L1 in tumor cells, intensifying the immunosuppressive capacity of Tregs and further suppressing the function of T cells.

### 5.2 Lactic Acid

In addition to the comparatively low oxygen content in the tumor microenvironment, tumor cells also undergo high levels of glycolysis, generating substantial amounts of lactic acid, which results in an acidic tumor microenvironment [103]. High lactic acid concentrations restrict lactic acid excretion from T cells, leading to increased lactic acid accumulation in these cells, which interferes with T cell metabolism and function, and inhibits anti-tumor immune responses in the TME [104]. Importantly, lactic acid-induced acidification increases arginase I expression in macrophages, limiting T-cell proliferation and activation [105]. Lactic acid inhibits the PI3K/Akt/mTOR pathway, thereby suppressing T cell glycolysis and enhancing FAO, inhibiting the differentiation of naive T cells to Teffs and promoting apoptosis of CD8^+^ tumor-infiltrating lymphocytes [106]. Lactate dehydrogenase B regulates pyruvate synthesis by controlling the ratio of nicotinamide adenine dinucleotide (NADH) to nicotinamide adenine dinucleotide (NAD), which attenuates T cell proliferation and inhibits aerobic glycolysis [107].

### 5.3 Immune Checkpoints

Tumors can activate the immune checkpoint (IC), a repressive signaling pathway in the immune system, which reduces immune cell activity and function while enhancing tumor cell proliferation [108, 109]. Recently, immune checkpoints research T cell such as PD-1 and cytotoxic T lymphocyte-associated protein 4 (CTLA-4), targeting T cells has made enormous strides in tumor therapy (Table **1**).

Recent studies have demonstrated that the activation of immune checkpoints is a crucial factor in immune escape during tumor progression [110]. Activation of immune checkpoints reprograms T-cell metabolism. The binding of PD-1/PD-L1 and increased production of CPT1 promote fatty acid oxidation, affect T cell glycolysis and glutamine catabolism, and inhibit the energy necessary for T cell activation and biosynthesis [111]. Furthermore, PD-1 activation could lead to a reduction in the number and length of mitochondrial cristae, altered structure and function, increased fatty acid oxidative utilization, reduced INF-γ production, and suppression of T cell function [109]. In contrast, alterations in mitochondrial structure may influence the expression of PD-1 and subsequently determine the fate of T cells [112]. T cell metabolic proliferation is suppressed upon activation, as cytotoxic T-lymphocyte–associated protein 4 (CTLA-4) inhibits downstream mTOR signaling by modulating Akt activation. Immune checkpoint inhibitors that target CTLA-4 facilitate the reactivation of T cell glycolysis and the production of interferon-gamma IFN-γ, thereby contributing to the reversal of immune tolerance [113].

## 6 TARGETED METABOLISM AGENTS COMBINED WITH ICIS

Immune checkpoint inhibitors (ICIs) have been widely used in the immunotherapy of various solid tumors, with low response rates and high rates of drug resistance being pressing issues [114]. Based on the tumor microenvironment, the combination of immune checkpoint inhibitors with agents that target the regulation of T-cell metabolic homeostasis could significantly improve the clinical results of immunotherapy. In this regard, the influence of nutrition metabolism on tumor immune escape has been discussed, along with the theoretical foundation for targeting nutrient metabolism in conjunction with PD-1/PD-L1 checkpoint blockade immunotherapy techniques (Table **2**) [115].

### 6.1 Targeted Metabolic Inhibitors Combined with ICIs

As an arginase inhibitor, CB-1158 alleviates the inhibition of T cell proliferation by MDSC *in vitro* and *in vivo*, and in combination with ICI increases the number of tumor-infiltrating CD8^+^ T cells [96]. CB-1158 alters the immune environment toward a pro-inflammatory state, inactivates myeloid cell-mediated immune escape, and reduces tumor growth. It is used both as a single agent and in combination with immune checkpoint inhibitors (ICIs) for the treatment of advanced or metastatic solid tumors [116]. As an inhibitor of the cholesterol esterase acetyl-CoA: acetyltransferase 1 (ACAT1), Avasimibe (Ava) enhances the effector function of T cells by increasing cell membrane cholesterol concentrations, which in turn promotes TCR aggregation. When combined with PD-1 inhibitors, it improves T-cell function and widens the therapeutic window of the combination [96].

IDO inhibitors alleviate amino acid deficiency and aberrant metabolite accumulation in the TME. Alternatively, the addition of certain amino acid metabolites, such as acetyl- CoA, spermidine, and creatine, can enhance the immune response of T cells. Notably, the IDO inhibitor indoximod is being tested in phase I/II clinical studies for metastatic melanoma in conjunction with PD-1 inhibitors or CTLA-4 inhibitors [117]. Although IDO inhibitors show promising prospects in clinical studies, further in-depth exploration and a relatively long research cycle are still necessary to verify their effectiveness and safety, thereby fully realizing their potential. PEGylated kynurenine (PEG-KYNase), an artificially tailored chronic enzyme, significantly suppressed the development of melanoma in mice. Moreover, the combination of PEG-KYNase with PD-1 inhibitors increased the response rate by 35% compared to PD-1 inhibitors alone [118]. As an IOD1 inhibitor, BGB-5777 enhanced Teffs function by antagonizing IDO1 and inhibiting tryptophan catabolism, thereby reducing the production of the metabolite KYN [119]. Meanwhile, the combination with a PD-1 monoclonal antibody can persistently improve the survival rate of patients with advanced glioblastoma. It is important to highlight that patients receiving treatment with PD-1 inhibitors in conjunction with IDO-1 inhibitors exhibit a notable risk of developing pneumonia and hepatitis. This risk is closely associated with the MHC class I allele within the HLA-B27 family, which is linked to autoimmune diseases [120]. In addition, the isocitrate dehydrogenase 1 inhibitor IDH305 suppresses the conversion of α-KG into the carcinogen metabolite D-2-hydroxyglutarate, thereby activating the mTOR signaling pathway, which has clinical potential in combination with immune checkpoint inhibitors for the treatment of patients with advanced solid tumors and gliomas [121].

CB-839 inhibits glutamine metabolism and biomolecule production as a renal-type glutaminase (GLS) inhibitor, which has a substantial inhibitory impact on esophageal squamous cell cancer [122]. In various clinical trials, CB-839, when combined with palbociclib, has shown a promising immune response and tolerability. V9302 significantly reduced glutamine absorption by tumor cells, increased the number of CD8^+^ cells in the tumor, and promoted CD8^+^ cell transfer from the tumor margin to the tumor core, while significantly killing 4T1 cells [123]. After V9302 was combined with anti-PD-L1, glutamine levels increased, and the proportion of activated T cells was significantly elevated in the tumor interstitial fluid. JHU083 blocked glutamine metabolism and inhibited glucose metabolism through the tricarboxylic acid cycle, glycolysis, and the pentose phosphate pathway (PPP), suppressing the immunosuppressive environment in the tumor microenvironment. This combination therapy can considerably strengthen the anti-tumor effects compared to PD-1 monotherapy [124].

### 6.2 Targeted Transduction Signal Inhibitors Combined with Icis

Small molecule medicines, in addition to directly controlling cellular metabolism, can be used in conjunction with immune checkpoint inhibitors to boost the immune system's anti-tumor capabilities *via* modifying signaling pathways. As reported [125], ADI-PEG 20 induced tumor T-cell infiltration in both a mouse melanoma model and a colon cancer model and inhibited tumor growth as a single agent or in combination with a PD-1 antibody. Everolimus, an mTOR inhibitor, increased PD-L1 expression in kidney cancer cell lines and, when combined with a PD-L1 monoclonal antibody, decreased RENCA proliferation [126]. The PPAR-α agonist bezafibrate enhances the expression of FAO-related genes, including PGC-1 and CPT1, in a Lewis lung cancer xenograft mouse model. It increases reactive oxygen species (ROS) levels and helps preserve FAO function and the activity of tumor-infiltrating lymphocytes (TILs). When combined with PD-L1 monoclonal antibody, it has a strong inhibitory effect on lung cancer [127]. Interestingly, the PDK1 inhibitor dichloroacetic acid (DCA), a nanoparticle that targets mitochondria and contains DCA prodrugs, exerts anti-tumor effects by altering TME and enhancing anti-tumor immune response in lung and liver cancer xenograft models of mice. When nanoparticles containing DCA precursors are combined with immune checkpoint inhibitors, the number of TIL cells increases significantly, and their immune cytotoxic activity is enhanced [128].

## 7 ADOPTIVE CELL THERAPY REGULATED BY T CELL METABOLISM *IN VITRO* COMBINED WITH ICIS

ICIs have partially addressed the challenge of chimeric antigen receptor T (CAR-T) cell exhaustion induced by the TME. The TME of solid tumors is characterized by conditions, such as hypoxia, elevated lactate levels, adenosine accumulation, and the presence of inhibitory cytokines. These factors contribute to mitochondrial dysfunction, abnormal glycolysis, and restricted oxidative phosphorylation in CAR-T cells, ultimately leading to their functional exhaustion [129, 130]. Research has shown that CAR-T cell exhaustion is closely linked to the activation of the PD-1/PD-L1 pathway, where high PD-1 expression inhibits the proliferation and cytokine release of CAR-T cells [131]. CAR-T cells with the PD-1 gene knocked out have exhibited enhanced persistence and tumor-killing efficacy in murine liver cancer models [132]. Preclinical studies have demonstrated that the anti-PD-1 agent pembrolizumab can significantly reduce the expression of exhaustion markers, such as T-cell immunoglobulin and mucin-domain containing-3 and lymphocyte-activation gene 3 in CAR-T cells, thereby promoting their infiltration into tumor sites [133]. Furthermore, from a metabolic regulation perspective, the delivery of IL-15 mRNA *via* lipid nanoparticles to enhance the energy metabolism of CAR-T cells has been shown to synergize with ICIs, thereby improving therapeutic efficacy [134].

Compared to CAR-T cells, TCR-T cells exhibit a broader targeting range by recognizing tumor antigens presented by major histocompatibility complex molecules. However, they are also susceptible to limitations, such as cellular exhaustion and immune escape. The exhaustion of TCR-T cells is intricately linked to the hypoxia-induced upregulation of CD39 and the activation of the PD-1/PD-L1 pathway within the TME [135]. Recent research has demonstrated that the fusion of 41BB and CD28 costimulatory molecules with the extracellular domain of PD-1, utilizing gene editing technology to construct the PD1-41BB CPS as a switch protein, enables TCR-T cells to initiate activation signals rather than inhibitory signals upon binding to PD-L1, thereby counteracting the immunosuppressive effects of the TME [136, 137]. Clinical studies have demonstrated that combining ICIs, such as anti-CTLA-4 antibodies, with TCR-T cells can substantially enhance their reactivity to tumor neoantigens. For instance, when TCR-T cells targeting the cancer germline antigen NY-ESO-1 are employed in the treatment of melanoma, an observed increase in the secretion of IFN-γ in tumor-infiltrating lymphocytes of patients is accompanied by a reduction in the expression of depletion markers [138]. Moreover, the synergistic application of rabbit myxoma virus and TCR-T cells has been shown to augment T cell infiltration by modulating the TME and facilitating the secretion of chemokines, including CXCL10 [139]. From a metabolic engineering standpoint, the overexpression of IL-21 or IL-15 can further mitigate the exhaustion of TCR-T cells, thereby enhancing their efficacy in conjunction with ICIs to establish sustained anti-tumor immunity [140].

## CONCLUSION

In summarize, metabolic abnormalities occur throughout carcinogenesis and progression, resulting in nutritional insufficiency in the TME. Additionally, immunological diseases and an immunosuppressive microenvironment are caused by T-cell metabolic imbalance. Elucidating the interplay between tumor metabolism and immune function has enhanced the efficacy of T-cell-mediated immunotherapy, providing a unique paradigm for clinical studies. To realize the full potential of combination therapy, it is critical to understand the processes of T cell metabolism and immunological activity. As a result, greater attention and effort should be focused on the metabolic mechanism of T-cell phenotypic alterations, as well as the discovery of immunometabolism checkpoints for improved therapeutic applicability.

Immune escape is inextricably linked to availability and T-cell activity in the tumor microenvironment. Notably, ICIs are frequently used in the immunotherapy of many solid malignancies. However, the clinical effectiveness of ICIs is limited by a lower response rate and a higher risk of intolerance. Future research should focus on the development of molecularly targeted drugs that modulate tumor and T cells within the TME to enhance the efficacy of immunotherapy. Cellular metabolism-targeted drugs might enhance therapeutic effectiveness in conjunction with ICIs by increasing T cell longevity and function in the TME. Furthermore, the combination of ICIs with nano-delivery systems has shown promise in overcoming T-cell exhaustion within the TME. These systems can modulate immune checkpoints and deliver therapeutic agents directly to the tumor site, reducing the required dosage and minimizing systemic toxicity [141]. Nanocarriers have been widely used in metabolism-mediated tumor treatment due to their accurate targeting, responsive release, and low toxicity. As a result, a major effort should be made to create nanocarriers that transport both tumor- and immune cell metabolism-specific medicines, thereby broadening the spectrum of immunotherapy for malignancies.

## Figures and Tables

**Fig. (1):**
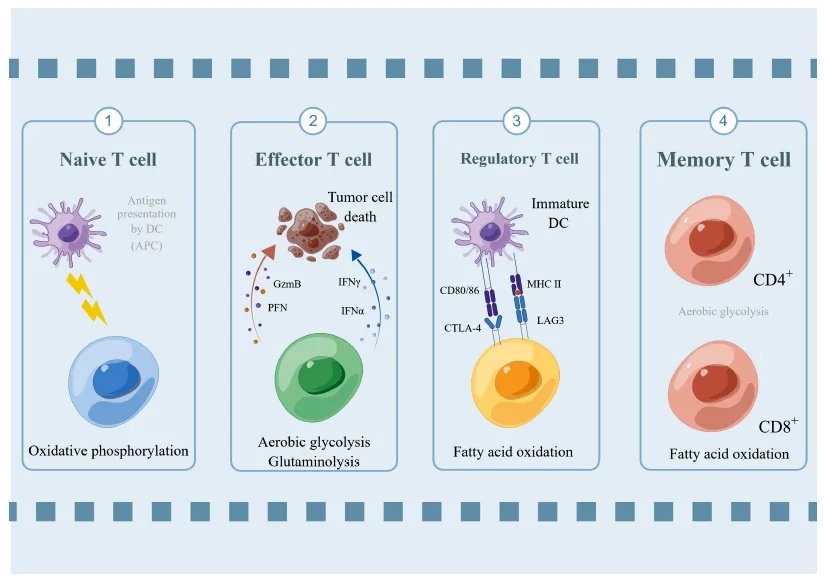
Characteristics of T cells in the tumor microenvironment. Various factors may affect the diversity and function of T cells, resulting in critical roles in tumor development and metastasis. Fatty acid metabolism and oxidative phosphorylation are vital for the proliferation and survival of naive T cells. The proliferation of activated effector T cells (Teffs) is heavily dependent on glycolytic metabolism even under aerobic conditions. Whereas regulatory T cells (Tregs) and memory T cells rely mostly on fatty acid oxidation.

**Fig. (2):**
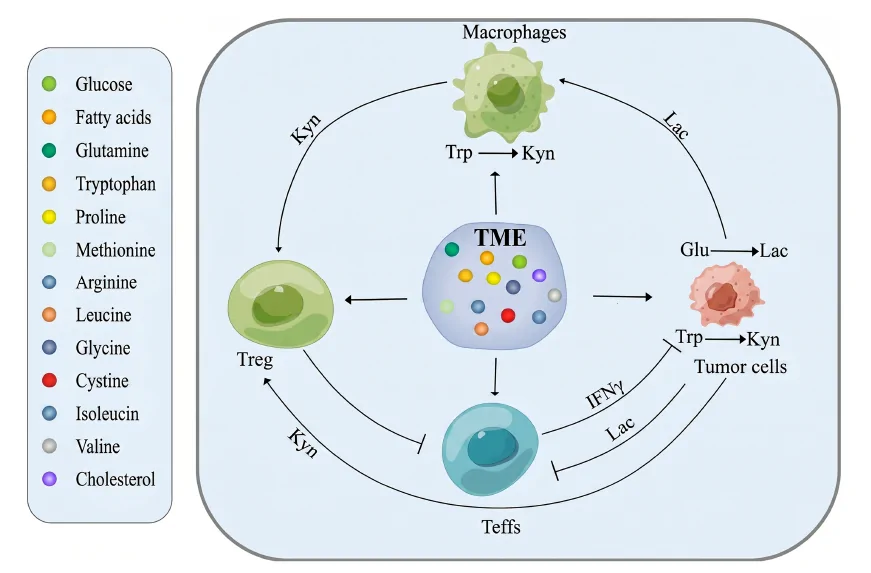
The role of nutrients and metabolism in the tumor microenvironment. Metabolic competition interferes with the activation, proliferation and differentiation of T cells, resulting in immune evasion.

**Fig. (3):**
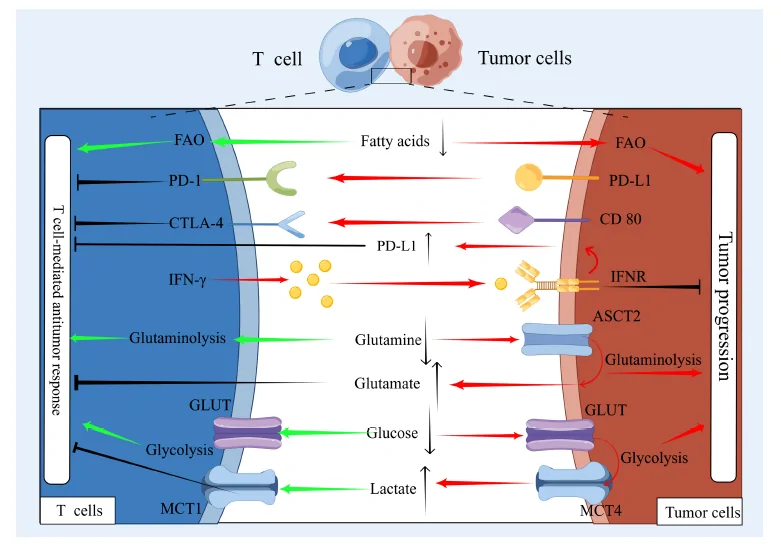
The tumor cells metabolites and immune checkpoints act a vital role in T cells metabolism-mediated immune responses.

**Table 1:** The effect of immune checkpoints on T cell function.

**Immune Checkpoint**	**Effects on Glucose Uptake**	**Effect on Mitochondrial Adaptation**	**Effect on Oxidative Stress**	**Effects on T Cell Function**	**References**
PD-1	Inhibition of glycolysis and glutamine decomposition	Damage to mitochondrial biosynthesis and function	Increase ROS accumulation	Inhibit T cell activation and promote T cell exhaustion	[105]
CTLA-4	Reduce glucose uptake and glycolysis	Reduce mitochondrial metabolic efficiency	Promote oxidative stress	Inhibit T cell activation	[110]

**Table 2:** Metabolic-targeted agents combined with ICIs.

**Agents**	**Targets**	**Tumor Types**	**Progress**	**Immune Checkpoint**	**References**
CB-1158	ARG1	Breast cancer	Phase I/ II	-	[113]
Avasimible	ACAT1	Brain cancer	Basic research phase	PD-1	[114]
Indoximod	IDO	Melanoma	Phase I/ II	PD-1 or CTLA-4	[115]
PEG-KYNase	KYN	Melanoma	Phase I/ II	PD-1	[116]
BGB-5777	IDO1	Glioblastoma	Basic research phase	-	[96]
IDH305	IDH1	Acute myeloid leukemia	Basic research phase	α-KG	[118]
CB-839	GLS1	Esophageal squamous cell carcinoma	Phase I/ II	-	[119]
V9302	ASCT2	Breast cancer	Basic research phase	PD-1	[120]
JHU083	Glutaminase	Colon cancer	Basic research phase	PD-1	[121]
ADI-PEG 20	CAT-1	Colon cancer	Phase I	PD-1	[122]
Everolimus	mTOR	Renal cell carcinoma	Basic research phase	PD-1	[123]
Bezafibrate	PGC-1α	Lung cancer	Basic research phase	PD-1	[124]
Dichloroacetate	PDK1	Colon cancer	Basic research phase	-	[125]

## LIST OF ABBREVIATIONS

ACAT1: Acyl Coenzyme A: Cholesterol Acyltransferase-1
ACLY: ATP-Citrate Lyase
AHR: Aryl Hydrocarbon Receptor
APCs: Antigen Presenting Cells
Ava: Avasimibe
Bax: BCL2 Associated X Protein
CAR-T: Chimeric Antigen Receptor T
CPT1α: Carnitine Palmitoyl Transferase 1α
CTLA-4: Cytotoxic T-Lymphocyte-Associated Protein 4
DCA: Dichloroacetic Acid
FAO: Fatty Acid Oxidation
FoxP3: Forkhead Box P3
HIF-1α: Hypoxia-Inducible Factor-1α
HK2: Hexokinase 2
IC: Immune Checkpoint
ICIs: Immune Checkpoint Inhibitors
IDO: Indoleamine 2,3-Dioxygenase
IFN-γ: Interferon-Gamma
MDSCs: Myeloid-Derived Suppressor Cells
NAD: Nicotinamide Adenine Dinucleotide
NADH: Reduced Form Of Nicotinamide-Adenine Dinucleotide
NO: Nitric Oxide
OXPHOS: Oxidative Phosphorylation
PD-1: Programmed Cell Death Protein-1
PD-L1: Programmed Cell Death Ligand-1
PEG-KYNase: PEGylated Kynureninase
PPP: Pentose-Phosphate Pathway
SAM: S-Adenosyl Methionine
scRNA-seq: Single-Cell RNA Sequencing
TCRs: T Cell Receptors
TDO: Tryptophan-2,3-Dioxygenase
Teff: Effector T Cells
Th1: T Helper Type 1 Cell
TME: Tumor Microenvironment
Tm: Memory T Cells
Tn: Naïve T Cells
Treg: Regulatory T Cells
scRNA-seq: Single-Cell RNA Sequencing
